# Prioritizing core areas, corridors and conflict hotspots for lion conservation in southern Africa

**DOI:** 10.1371/journal.pone.0196213

**Published:** 2018-07-05

**Authors:** Samuel A. Cushman, Nicholas B. Elliot, Dominik Bauer, Kristina Kesch, Laila Bahaa-el-din, Helen Bothwell, Michael Flyman, Godfrey Mtare, David W. Macdonald, Andrew J. Loveridge

**Affiliations:** 1 US Forest Service, Rocky Mountain Research Station, Flagstaff, AZ, United States of America; 2 Wildlife Conservation Research Unit, Department of Zoology, University of Oxford, The Recanati-Kaplan Centre, Tubney House, Tubney, Oxon, United Kingdom; 3 Department of Animal Ecology and Conservation, University of Hamburg, Hamburg, Germany; 4 School of Life Sciences, University of KwaZulu-Natal, Durban, South Africa; 5 Northern Arizona University, Department of Biology, Flagstaff, AZ, United States of America; 6 Department of Wildlife and National Parks, Gaborone, Botswana; 7 KAZA TFCA Secretariat, Zimbabwe Country Office, Victoria Falls, Zimbabwe; University of Tasmania, AUSTRALIA

## Abstract

Conservation of large carnivores, such as the African lion, requires preservation of extensive core habitat areas, linkages between them, and mitigation of human-wildlife conflict. However, there are few rigorous examples of efforts that prioritized conservation actions for all three of these critical components. We used an empirically optimized resistance surface to calculate resistant kernel and factorial least cost path predictions of population connectivity and conflict risk for lions across the Kavango-Zambezi Transfrontier Conservation Area (KAZA) and surrounding landscape. We mapped and ranked the relative importance of (1) lion dispersal areas outside National Parks, (2) corridors between the key areas, and (3) areas of highest human-lion conflict risk. Spatial prioritization of conservation actions is critical given extensive land use redesignations that are reducing the extent and increasing the fragmentation of lion populations. While our example focuses on lions in southern Africa, it provides a general approach for rigorous, empirically based comprehensive conservation planning based on spatial prioritization.

## Introduction

Africa’s human population is expected to increase threefold by the end of this century [1], with highest population growth near protected areas [2]. Rising human and livestock populations increase demand for resources, driving conversion of land currently protected for wildlife to uses, such as agriculture or mining, that are perceived to be more economically profitable or politically expedient [3, 4]. Accelerating declines of carnivores as a result of human-carnivore conflict and habitat loss are likely [5].

In the case of the African Lion (*Panthera leo*), human-wildlife conflict and habitat loss are the primary drivers of recent declines, with lion populations within protected areas becoming increasingly isolated [6]. Bjorklund [7] found that a minimum of 50–100 prides, linked by dispersal, is required to maintain long-term genetic diversity. Very few remaining populations contain this number of prides [6]) and some populations already have reduced genetic diversity, which has been shown to decrease reproductive performance [8] and increase susceptibility to disease [9]. Many existing protected areas are too small to support large populations and are therefore unlikely to be viable in the long term. Dispersal between remnant populations is therefore critical in maintaining long-term genetic health and providing demographic rescue of regional lion populations [10] and inbreeding depression [8,9]. In response to growing conservation concern, similar to those introduced above for lions, the creation or protection of dispersal corridors has emerged as a popular strategy to improve population connectivity and enhance viability [11, 12, 13].

The Kavango-Zambezi Transfrontier Conservation Area, KAZA (~520,000 km^2^), in southern Africa is of immense conservation importance for lions as it contains 13 ‘Lion Conservation Units’ [14], including the Okavango-Hwange population, one of Africa’s 10 remaining lion ‘strongholds’ [15]. In this paper, we present a comprehensive approach to prioritizing lion conservation actions based on spatial optimization with empirical connectivity models. We assess the importance and vulnerability of the key dispersal areas (defined as areas outside national parks and game reserves with high dispersal value for lions and therefore the overall integrity of lion range) and movement corridors between them, and identify areas of high human-lion conflict risk across the KAZA landscape.

## Materials and methods

The project involved the use of data on lion movements obtained from GPS collars. All lion handling, collaring and research was approved under stringent protocols and approvals granted by the University of Oxford, and all lion handling was done by a professional and certified wildlife veterinarian.

### Resistance surface

**Fig 1:**
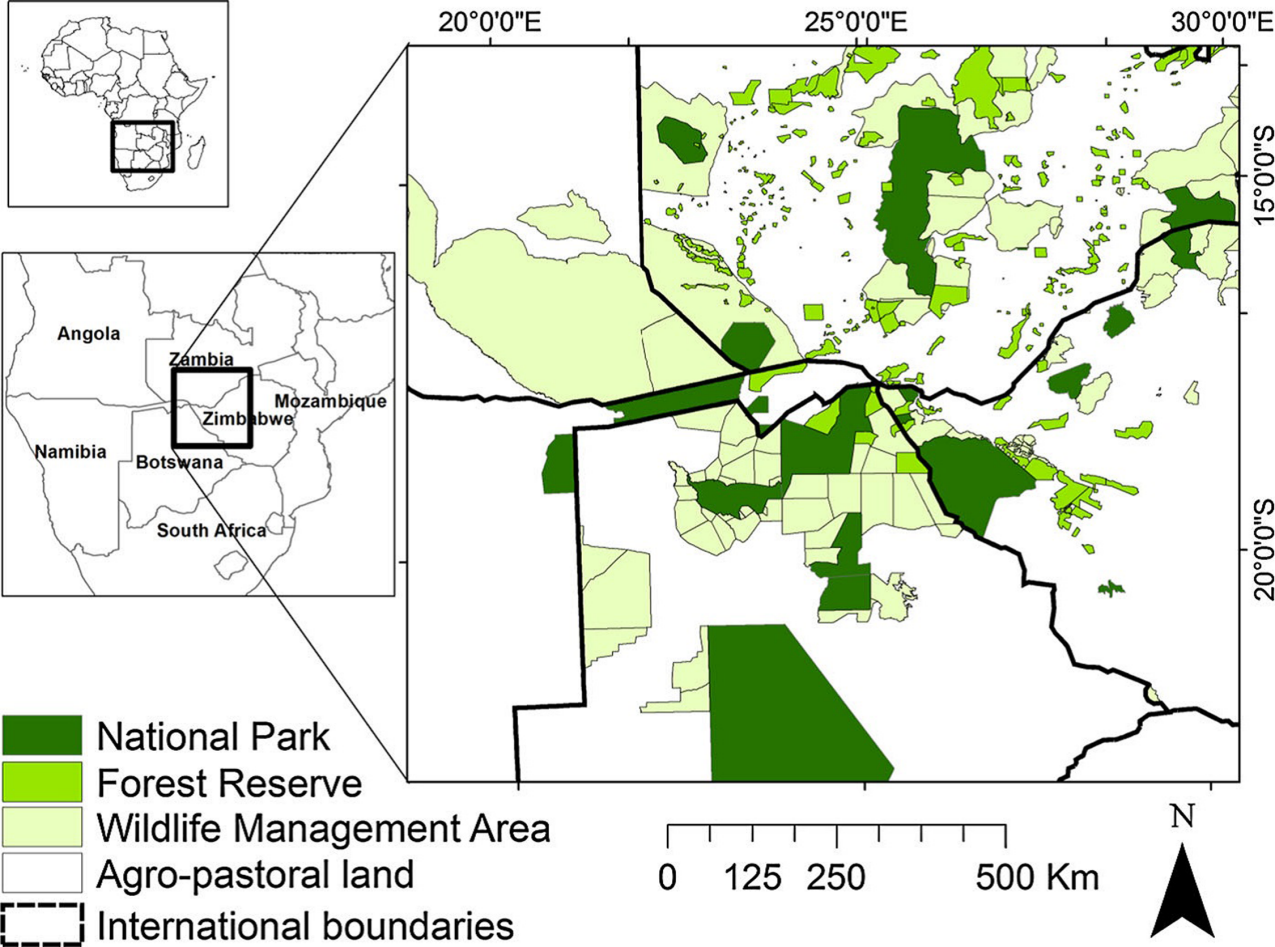
Study area orientation map. Top left shows study area extent within the African continent; bottom left shows study area extent within southern Africa, with inset of land-uses in the study area.

We used a resistance surface that was empirically optimized for the study extent using Global Positioning System data collected from male natal dispersers (Fig 1; for a full description of the study extent and resistance surface modelling, see[16]. Briefly, Elliot et al. [16] used a multi-scale, path-selection function to predict landscape resistance based on movement data for adult female, adult male and dispersing juvenile male lions. Given that juvenile dispersal is disproportionately important in population connectivity we use the resistance surface produced by [16] for dispersing juvenile males in this analysis. This surface was produced with a 500m pixel size, and predicted that juvenile males selected movement paths preferentially in protected areas and avoided communal lands, proximity to towns, areas with high human population density, and large roads.

### Source points for connectivity modeling

The connectivity modeling approaches used in this study (resistant kernel and factorial least cost path) are based on predicting movement from a set of source points that reflect the distribution and density of the underlying population. Source points were established by intersecting the protected area extent with a map of estimated site carrying capacity for lions based on climatic correlates of prey biomass [17]. Specifically, we down sampled the predicted density based on carrying capacity to reflect the low densities of lions outside of protected areas and also adjusted in areas where we had first-hand knowledge that the lion population differed from the prediction [17] (e.g., Angola). We converted these densities to source points for connectivity modeling following a three-step process. First, we rescaled the density layer such that it reflected the probability of a lion occurring in each 500m pixel. Second, we took the product of the rescaled density layer and a raster layer of uniform random values between 0 and 1. Third, we selected all values of the product that were positive as source points, producing a selection of source points that matches the density predicted by the density surface.

### Resistant kernel and factorial least cost path modeling

The resistance map provides resistance values for all locations in the study extent, in the form of the cost of crossing each pixel relative to the least-cost condition [16]. We used UNICOR [18] to calculate cumulative resistant kernel and factorial least cost path maps. We specified a dispersal threshold of 1,000,000 cost units for the resistant kernel analysis [19]. We calculated the factorial least cost path network without a dispersal threshold (as in [19, 20]) to provide a broad scale assessment of the regional pattern of potential linkage.

The factorial least cost path analysis calculates the least cost paths among all combinations of source points and sums them to create a path density map reflecting the relative strength of linkage across the network. The resistant kernel model calculates the expected movement of dispersing lions in each pixel, given the dispersal ability of the species and the resistance of the landscape [21, 22]. The cumulative resistant kernel density can be interpreted as the probability of a dispersing lion traversing that pixel, given the location of the source points and the resistance of the landscape.

### Identifying key lion dispersal areas and linkage corridors

We defined key dispersal areas as contiguous patches of cumulative resistant kernel values greater than the 25^th^ percentile of the cumulative kernel surface. These reflect areas of moderate to high predicted movement rates. Our goal was to evaluate the importance of key lion areas (‘dispersal areas’) that were outside National Parks and Game Reserves, so we selected the cumulative kernel surface values outside of these strictly protected areas for analysis of the number and relative importance of these dispersal areas. Likewise, we selected linkage corridors that were greater than the 25^th^ percentile of the distribution of values in the factorial least cost path surface, and outside the network of National Parks/Game Reserves and dispersal areas, as identified above.

### Mapping relative conflict risk

We reasoned that areas with rapid change in cumulative kernel value (i.e. rapid changes from high to low expected dispersal rates) are potential hotspots where high lion movement intersects high conflict risk. We predicted conflict risk zones by calculating the standard deviation of the cumulative kernel surface within a 10km focal radius for all areas outside of National Parks/Game Reserves, and selected values above the 25^th^ percentile for further analysis. Essentially, this index calculates the spatial variation in local cumulative kernel value, identifying areas with high change that we would expect to represent areas with high relative conflict risk.

### Evaluating the relative importance of predicted key lion dispersal areas, linkage corridors and conflict hotspots

**Fig 2:**
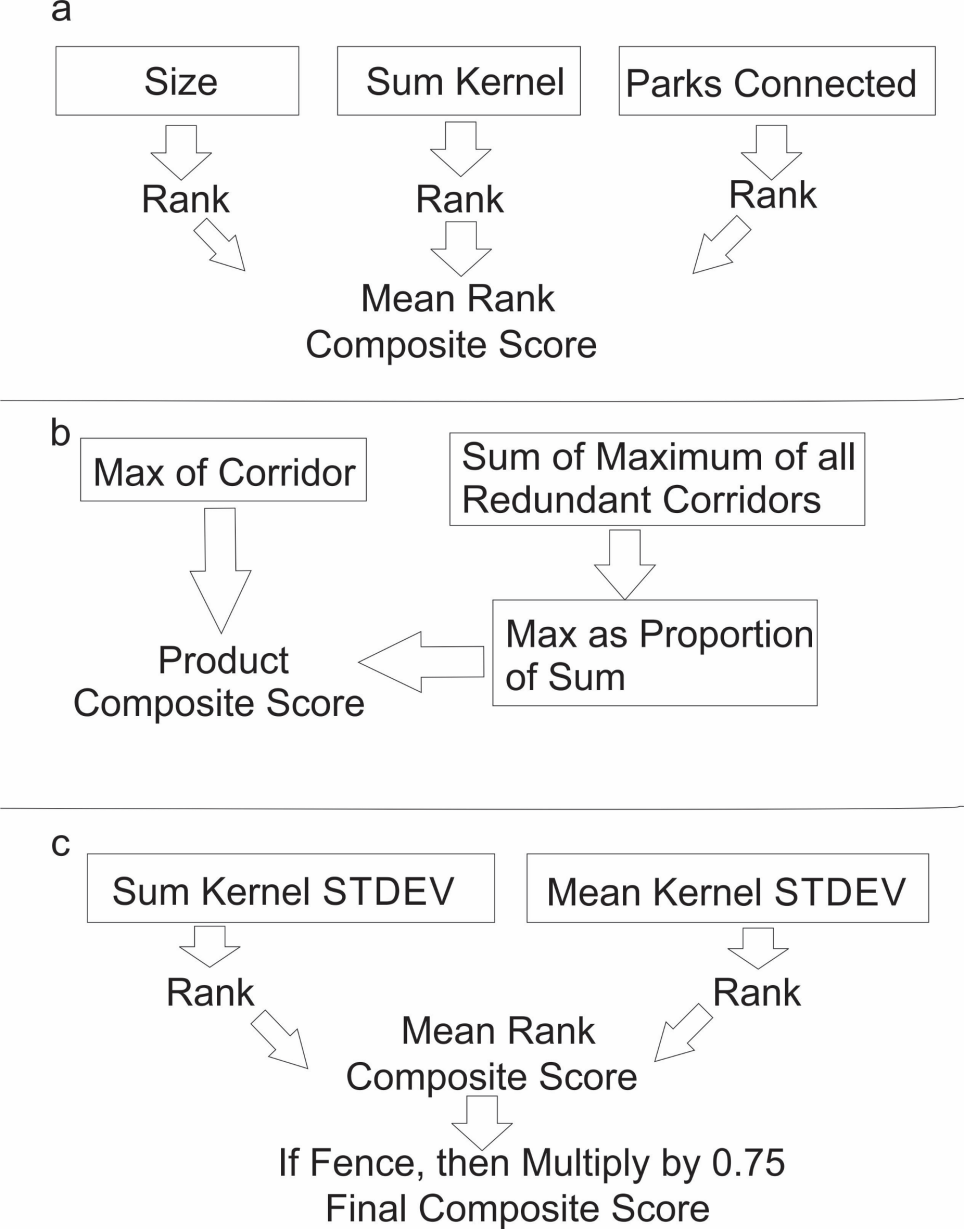
Schematic of ranking. Steps to produce composite ranks for (a) core areas, (b) corridors, and (c) conflict hotspots.

We used several criteria to evaluate the importance of predicted dispersal areas, linkage corridors, and conflict hotspots. For dispersal areas, we identified three main characteristics that contribute to their importance to regional lion populations: (1) The size of the area, since lion populations require large areas; (2) The summed kernel value, reflecting the total predicted movement of dispersing lions in that region of the landscape; (3) The degree to which a key dispersal area was connected to other areas, because dispersal areas that are nodes connecting the regional populations are likely more important than peripheral populations (Fig 2A). We therefore produced a measure of dispersal area importance based on number of National Parks/Game Reserves it connected. We produced a composite score by averaging the ranks produced by these three measures.

We used two measures to evaluate the importance of predicted linkage corridors (Fig 2B). (1) We extracted the maximum value of the factorial least cost path surface for each linkage corridor segment. This reflects corridor strength (*sensu* [23]) in terms of the number of pairwise linkages between source points predicted to traverse that corridor segment. (2) We weighted linkage corridor strength as a function of corridor redundancy. Specifically, a corridor between two dispersal areas that have no other linkage between them is more important than a corridor linking another two dispersal areas that are also linked by other corridors. We calculated corridor redundancy for each corridor by calculating the proportion of total connectivity (as measured by the sum of maximum corridor strength measures across all linkages between the two dispersal areas joined by the focal corridor) that is provided by the focal corridor. We produced a composite linkage that is the product of these two measures of corridor importance. Linkage corridors that are individually strong and have low redundancy are weighted highest, while those that have low strength and multiple alternative corridor routes are weighted lowest in importance.

Finally, we produced two measures of the importance of predicted conflict hotspots (Fig 2C): (1) We measured conflict hotspot strength based on the sum of the kernel standard deviation surface within each identified patch of predicted high conflict risk. This measure weights areas based on total conflict risk and is highly dependent on the area of the predicted conflict hotspot; (2) We calculated the mean value of the kernel standard deviation surface within each predicted conflict hotspot patch. This measure identifies areas of highest potential conflict risk, regardless of size. For management purposes, both of these measures are informative, and we combined them with equal weight; (3) We multiplied the conflict hotspot combined value by 0.75 if it was traversed by a fence since conflict hotspot patches that coincide with the location of a wildlife fence likely have reduced conflict risk since the fence partly separates people and cattle from lions, and also because the fence itself, as a resistant feature in the resistance model, contributes to the high values of the kernel standard deviation surface.

## Results

### Location and importance of key dispersal areas

**Fig 3:**
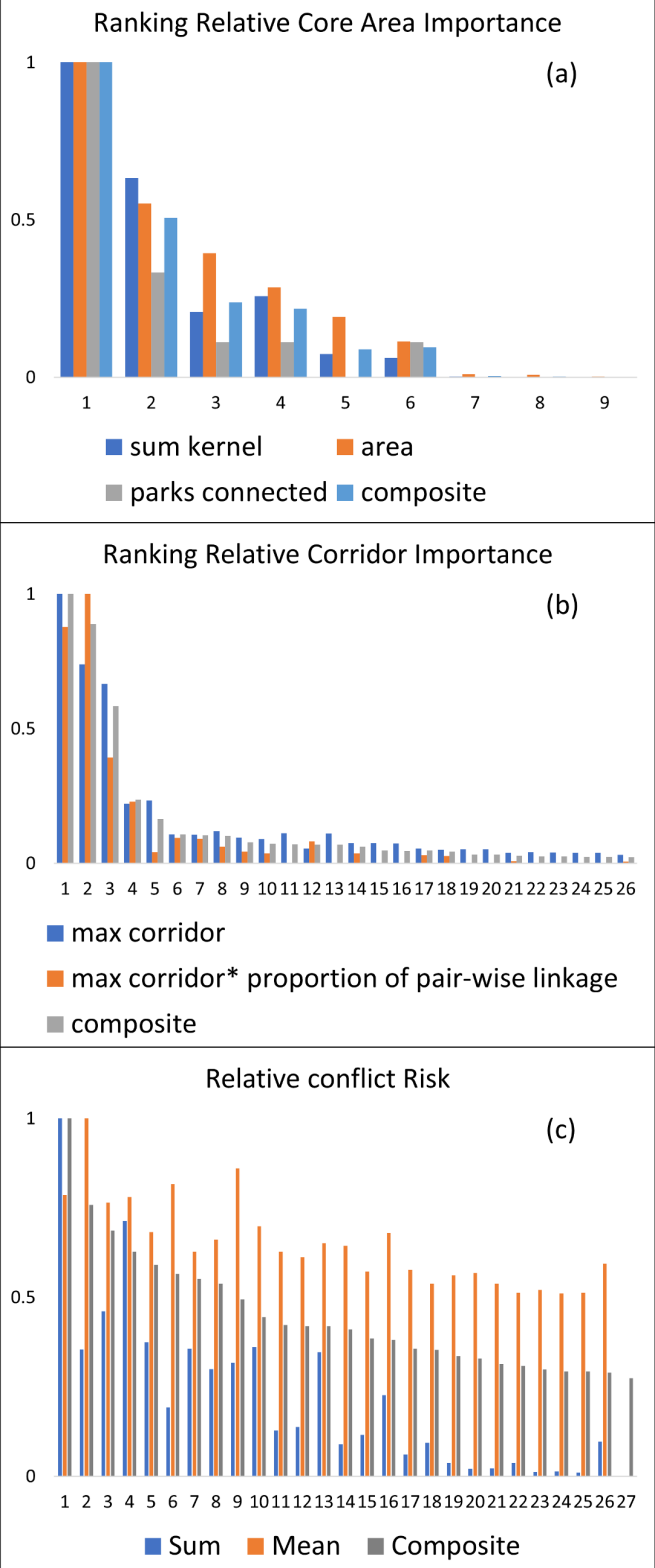
Relative importance rankings. (a) key lion dispersal areas, (b) lion linkage corridors, (c) human-lion conflict risk in the Kavango-Zambezi Transfrontier Conservation Area and surrounding landscape. Numbers refer to labels in Figs 4, 5 and 6.

**Fig 4:**
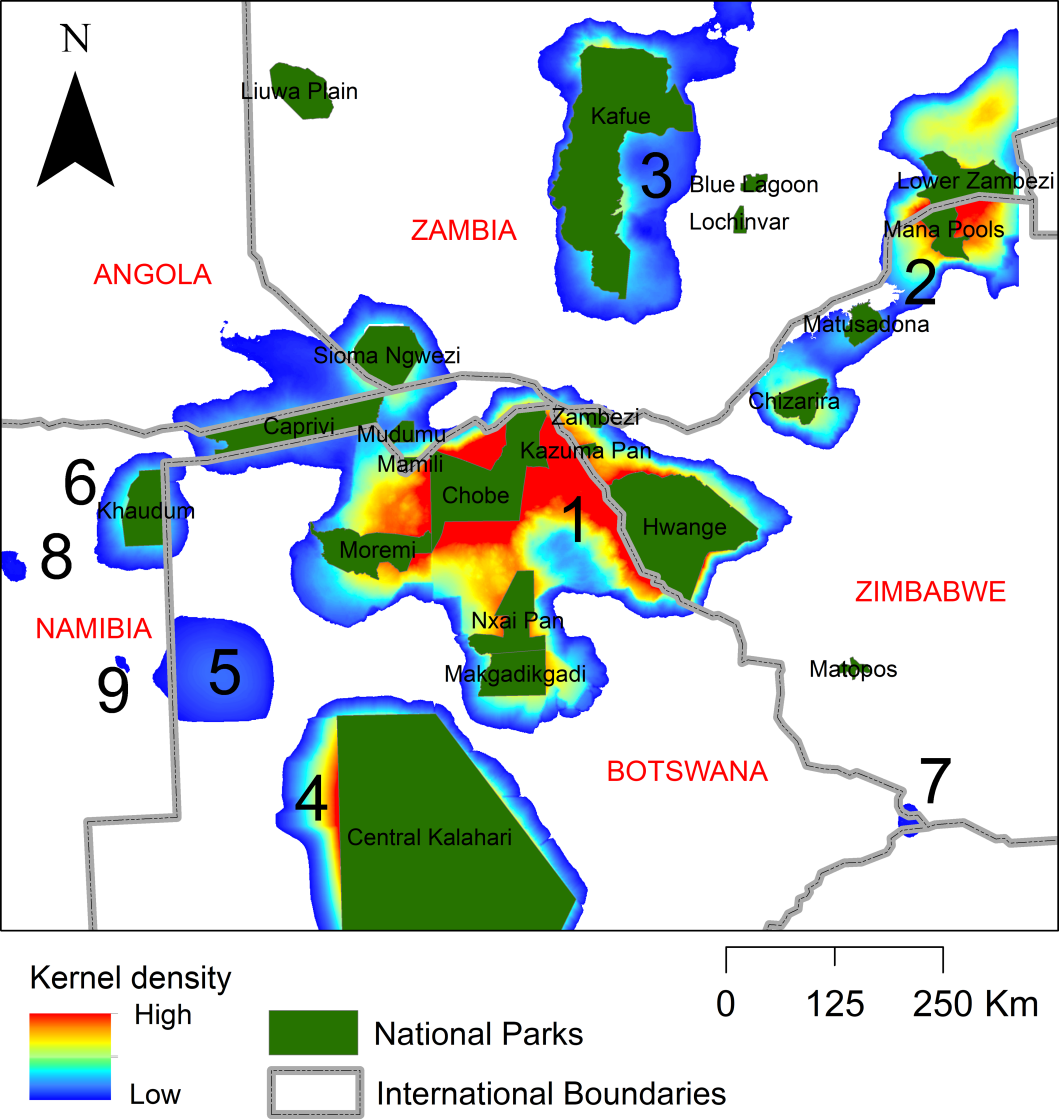
Dispersal areas. Ranked values of composite lion dispersal area importance within the Kavango-Zambezi Transfrontier Conservation Area and surrounding landscape.

We identified nine key dispersal areas, which differed dramatically in predicted strength (Figs 3A and 4). Based on the scree-plot of relative importance ranking (Fig 3A) we selected four key dispersal areas to emphasize. Dispersal area 1, ranked as by far the most important, surrounds and connects Chobe, Nxai Pan, Moremi, Hwange, and Makgadikgadi Pans protected areas. The second most important dispersal area connected the protected areas of Chete, Chizarira, Chirisa, Charara, Mana Pools, Chewore, Doma in Zimbabwe and Lower Zambezi in Zambia. This dispersal area had a composite importance score of 50.6% of the highest ranked dispersal area. The third ranked dispersal area surrounded Kafue National Park in Zambia. The composite importance measure for this predicted dispersal area was 23.7% of the highest ranked dispersal area. The fourth ranked dispersal area surrounded the Central Kalahari Game Reserve, particularly concentrated on the western boundary, with a composite score of 21.6% of the highest ranked dispersal area.

### Location and importance of predicted linkage corridors

**Fig 5:**
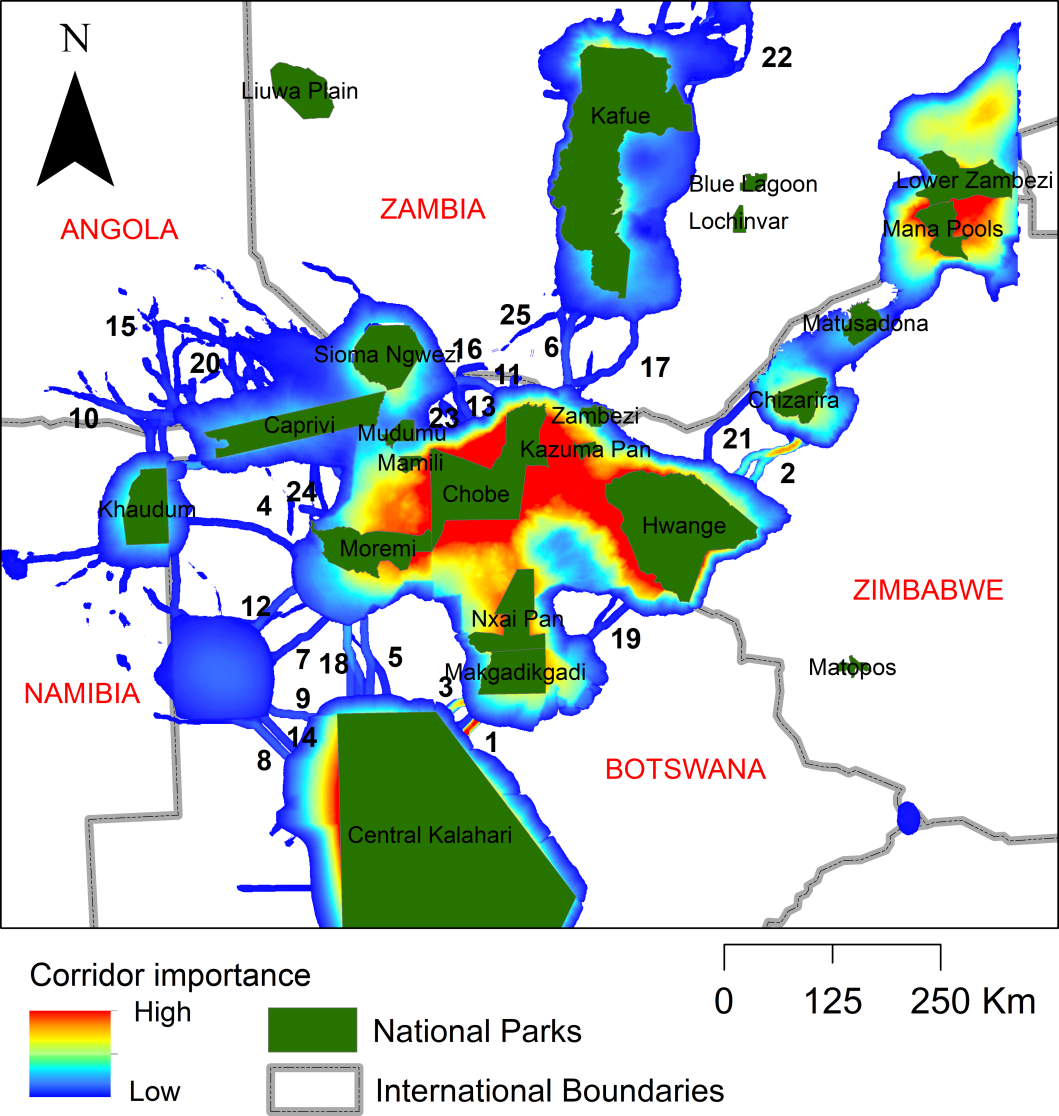
Corridors. Ranking of relative lion linkage corridor importance within the Kavango-Zambezi Transfrontier Conservation Area and surrounding landscape.

We predicted 27 linkage corridors between the nine key dispersal areas (Fig 5), which differed greatly in strength and relative importance. Based on the scree plot of composite ranking (Fig 3B) we selected three linkage corridors to emphasize. The highest ranked corridor was located between the southwestern corner of the central (highest ranking) dispersal area and the dispersal area surrounding the Central Kalahari Game Reserve, proximally linking Makgadikgadi Pans National Park and Central Kalahari Game Reserve. The second highest ranked corridor was located between the northeastern corner of the central dispersal area and the southwestern corner of the second highest ranked dispersal area, proximally linking Hwange and Chizarira National Parks. This corridor had a relative composite value of 88.8% of the highest ranked corridor. The third highest ranked corridor was near and parallel to the first, between the Makgadikgadi Pans and Central Kalahari protected areas, and had a composite value of 58.4% of the highest ranked corridor.

### Location and importance of predicted conflict hotspots

**Fig 6:**
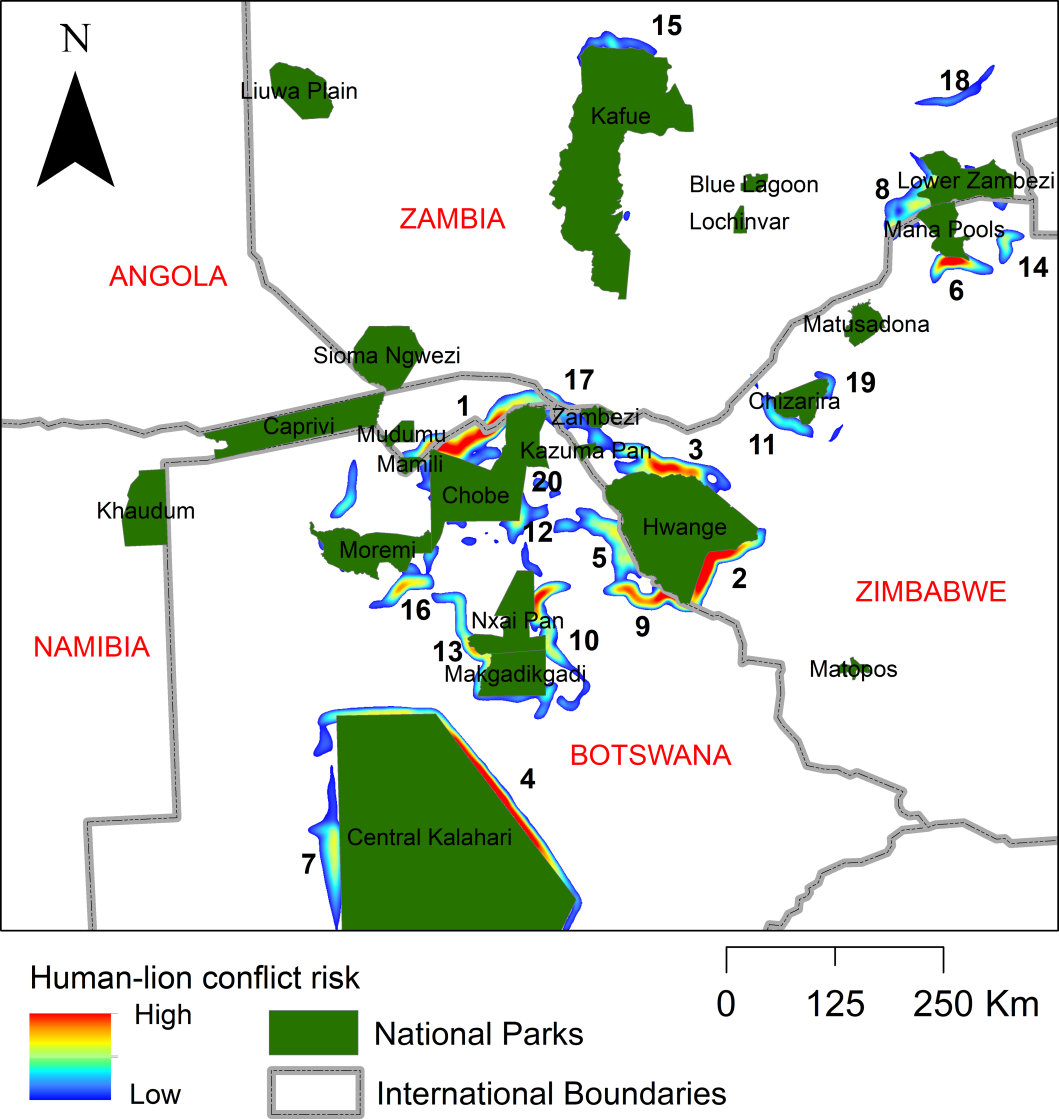
Conflict hot-spots. Ranking of relative human-lion conflict hotspot importance within the Kavango-Zambezi Transfrontier Conservation Area and surrounding landscape.

The highest ranked predicted conflict hotspot area runs along the northern edge of the central dispersal area from Mudumu National Park in the west to Zambezi National Park in the east, with an area of particularly intense predicted conflict within the Chobe Enclave north of Chobe National Park (Figs 6 and 3C). The second highest ranked conflict risk zone is along the eastern edge of the central dispersal area, running along the eastern boundary of Hwange National Park. This conflict hotspot had a composite score of 75.8% of the highest ranked conflict hotspot. The third highest ranked conflict hotspot was also adjacent to Hwange National Park in the central dispersal area, running along the northern boundary of the park, with a relative conflict risk value of 68.7% of the highest ranked conflict risk hotspot.

## Discussion

Habitat loss and fragmentation, coupled with severe human-wildlife conflict, have reduced lion populations to less than 10% of their historic range. It is widely recognized that conservation of lions, and other large carnivores, requires a combined strategy incorporating the preservation of extensive core habitat areas, linkages between them, and mitigation of human-wildlife conflict. However, there have been few rigorous examples of efforts that have spatially prioritized conservation actions for all three of these critical components. As human populations continue to grow [1], so too does demand for land, which is likely to exacerbate the two most pertinent threats facing lions, habitat loss and human-lion conflict [6]. It is therefore imperative that policy makers prioritize conservation actions based on the available scientific evidence. Our paper presents a comprehensive strategy for lion conservation across the Kavango-Zambezi Transfrontier Conservation Area in southern Africa, which combines a validated empirical connectivity model with spatial prioritization of core areas, corridors and conflict risk hotspots, to motivate directed and immediate conservation for this region. This is particularly critical at the present moment given rapidly increasing human populations leading to extensive land use redesignations that are reducing the extent and increasing the fragmentation of the lion population.

We identified nine key dispersal areas, 27 linkage corridors and 27 potential human-lion conflict hotspots outside National Parks in the KAZA Transfrontier Conservation Area and its surrounding landscape. Our results suggest that it is critical to ensure that Dispersal Areas 1, 2 and 3 continue to be managed for wildlife in their entirety. With four strategically placed corridors (Corridors 1, 2, 4 and 6; Figs 4 and 5), the five most important Dispersal Areas can be linked, and we urge that these be designated and enhanced, perhaps by establishing funneling fences to direct dispersers into them [24]. Finally, the four areas most at risk of human-lion conflict (Fig 6) require conservation action, either in the form of strategically placed fences, or mitigation measures, or both. In summary, our results suggest that the most effective means of maintaining the long-term viability of lions in this region is to maintain the current Protected Area network, protect the most important Dispersal Areas, protect and enhance Corridors 1, 2, 4, 5 and 6, and implement conflict mitigation measures in the areas most at risk.

While habitat loss and fragmentation are major drivers of lion declines, so too is human-lion conflict [6], and therefore human-lion conflict must be addressed in connectivity planning. Our analysis highlighted the area within the Chobe Enclave as being most at risk of conflict, while the Tsholotsho area, to the south-east of Hwange National Park was ranked second. The third area most at risk of conflict is located to the north-east of Hwange National Park, while the fourth is east of the Central Kalahari Game Reserve. All these areas are known conflict hotspots [25], and according to our least cost path analysis, offer little or no connectivity to other areas. We therefore suggest one of two courses of action to minimize human-lion conflicts in these hot spots. First, strategically placed fences could be erected to limit movement of lions into these specific areas, a measure which is unlikely to reduce connectivity in these particular locations [24]. A strong caveat to this solution is that fences must be predator proof and adequately maintained. The second alternative would be to implement community-based mitigation initiatives aimed at either reducing the levels of conflict or maximizing the incentives to protect wildlife. It is likely that the area east of the Central Kalahari Game Reserve experiences less conflict due to the fence in that area and we advocate that the fence is maintained and reinforced to minimize conflicts.

The analyses presented here focus on identifying the most important core areas, the strongest potential corridors that connect them, and the locations of the highest potential conflict risk. Our recommendation is to (1) protect the most important core areas, (2) establish movement across the most important corridors, and protect them from development and conflict risk, and (3) implement conflict mitigation measures and strategic fencing to reduce mortality risk to lions in the identified conflict hot spots. In some cases, it may no longer be possible to functionally restore some of the movement corridors identified in our analyses. In such cases, a potential alternative would be to mimic the outcomes that would result if the corridors were functional, such as translocations of individual lions reciprocally across the gap [26]. Translocations between fenced protected areas has been a successful strategy for maintaining lion genetic diversity, but where possible we advocate for establishing and protecting functional corridors, since functional corridors would provide connectivity for a large number of species, in addition to lions.

It is important for conservation proposals to include consideration of risks, hidden risks, opportunity costs and cost implementation. The prioritization presented here is based exclusively on ranking locations for conservation based on biological criteria only, and does not include discussion of societal, political, or economic considerations. As such, it should not be considered to be a recommendation for specific action, but rather a step in the process of decision-making. We believe these results will be useful to managers and decision-makers in their efforts to identify solutions that meet conservation and social objectives simultaneously in a cost-effective manner. Future research, combining sociology, economics and ecology, should work on formalizing that process of balancing the conservation and social objectives surrounding lion conservation in southern Africa.

This example, while for lions in southern Africa, provides a general approach for rigorous, empirically based comprehensive conservation planning based on spatial prioritization. We propose a method to quantitatively develop a comprehensive strategy for population-level carnivore conservation based on combining validated empirical connectivity models with spatial prioritization of core areas, corridors and conflict risk hotspots. By spatially mapping and ranking the relative importance of these areas our approach allows managers to identify the highest priority areas for directed and immediate conservation.

## Supporting information

S1 File Resistance layer used as the base of connectivity modeling reported in this paper. (RSG)

S2 File Source points representing locations of individual lions included in connectivity modeling reported in this paper. (XY)

S3 File Cumulative resistant kernel surface that is the product of UNICOR connectivity modeling on the resistance layer (S1 File) and source points (S1 File). (ADDEDPATHS)

S4 File Factorial least-cost path network surface that is the product of UNICOR connectivity modeling on the resistance layer (S1 File) and source points (S2 File). (ADDEDPATHS)
